# Ruptured postpartum ovarian artery aneurysm, endovascular limitations and the value of open vascular control

**DOI:** 10.1016/j.jvscit.2026.102317

**Published:** 2026-05-14

**Authors:** Max Murray-Ramcharan, Jenny Ramzi, Mina Iskaros, S. Christopher Frontario, Thomas Bernik

**Affiliations:** a Department of Vascular Surgery, Englewood Health, Englewood, NJ; b Department of Biological Sciences, New Jersey Institute of Technology, Newark, NJ

**Keywords:** Ovarian artery aneurysm, Postpartum hemorrhage, Rupture, Embolization, Major vascular ligation

## Abstract

Ruptured ovarian artery aneurysm (OAA) is an exceptionally rare cause of postpartum retroperitoneal hemorrhage and is often difficult to diagnose due to nonspecific presentation. We report a postpartum patient with intermittent symptoms whose computed tomography scan suggested ovarian artery bleeding, followed by an unsuccessful endovascular embolization attempt due to severe vessel tortuosity. She ultimately underwent open surgical exploration with ligation of the ruptured OAA, achieving definitive hemostasis and full recovery. This case highlights the diagnostic challenges of OAA rupture and underscores the importance of prompt operative intervention when endovascular management is not feasible.

Ovarian artery aneurysm (OAA) rupture is an exceptionally rare vascular emergency, with <50 cases reported in the literature.^1^ Although most described cases occurred during late pregnancy or in the immediate postpartum period, likely related to various hormonal changes, OAA rupture can present across a wider clinical spectrum and is often difficult to diagnose due to its nonspecific symptoms and overlap with more common causes of intra-abdominal bleeding.^2^^,^^3^

From a vascular surgery standpoint, OAA rupture represents a uniquely challenging source of retroperitoneal hemorrhage. Diagnosis is frequently delayed, because imaging findings can be subtle, and the condition is rarely considered early in the differential.^4^ Computed tomography (CT) angiography may identify contrast extravasation from the ovarian artery, but visualization can be limited by vasospasm, intermittent bleeding, or displacement by hematoma.^5^ Endovascular embolization has emerged as a preferred first-line option for stable patients; however, technical failure can occur due to tortuosity, small-caliber vessels, or difficulty catheterizing the ovarian artery.^6^ In such cases, or in patients with ongoing hemodynamic instability, prompt surgical exploration and direct vascular control remain essential and can be lifesaving.^7^

We present a case of postpartum retroperitoneal hemorrhage due to a ruptured OAA that was initially suspected on imaging, underwent attempted endovascular management, and ultimately required open surgical repair after failed embolization. This case highlights the diagnostic complexity of this rare vascular entity and the importance of a flexible, multimodal approach to management. The patient provided written consent for case details and images to be published.

## Case report

**Fig 1:**
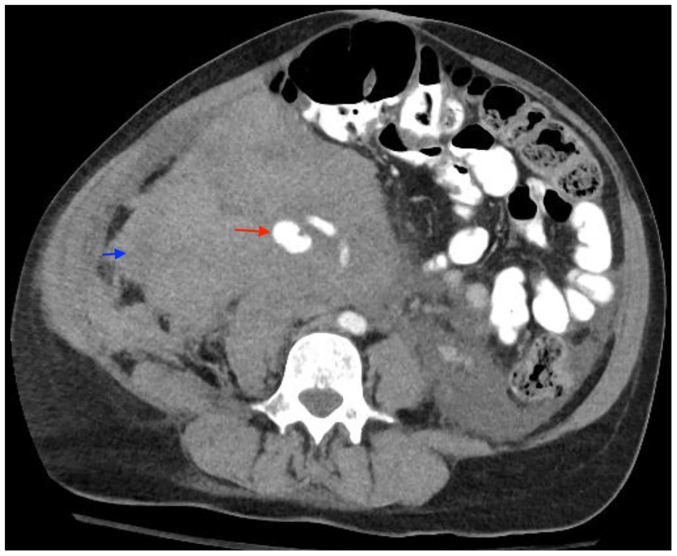
Computed tomography (CT) scan demonstrating right ovarian artery aneurysm (OAA) (*red arrow*) and associated retroperitoneal hematoma (*blue arrow*).

**Fig 2:**
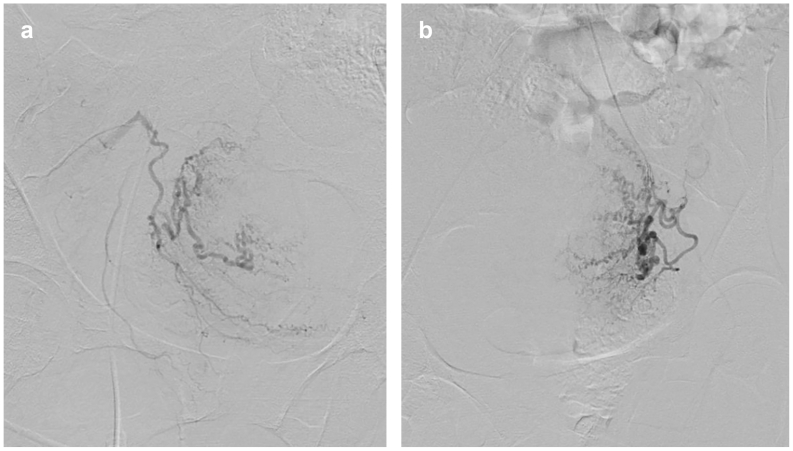
**(A)** Left uterine artery prior to embolization, no extravasation present. **(B)** Right uterine artery before embolization, no extravasation present.

**Fig 3:**
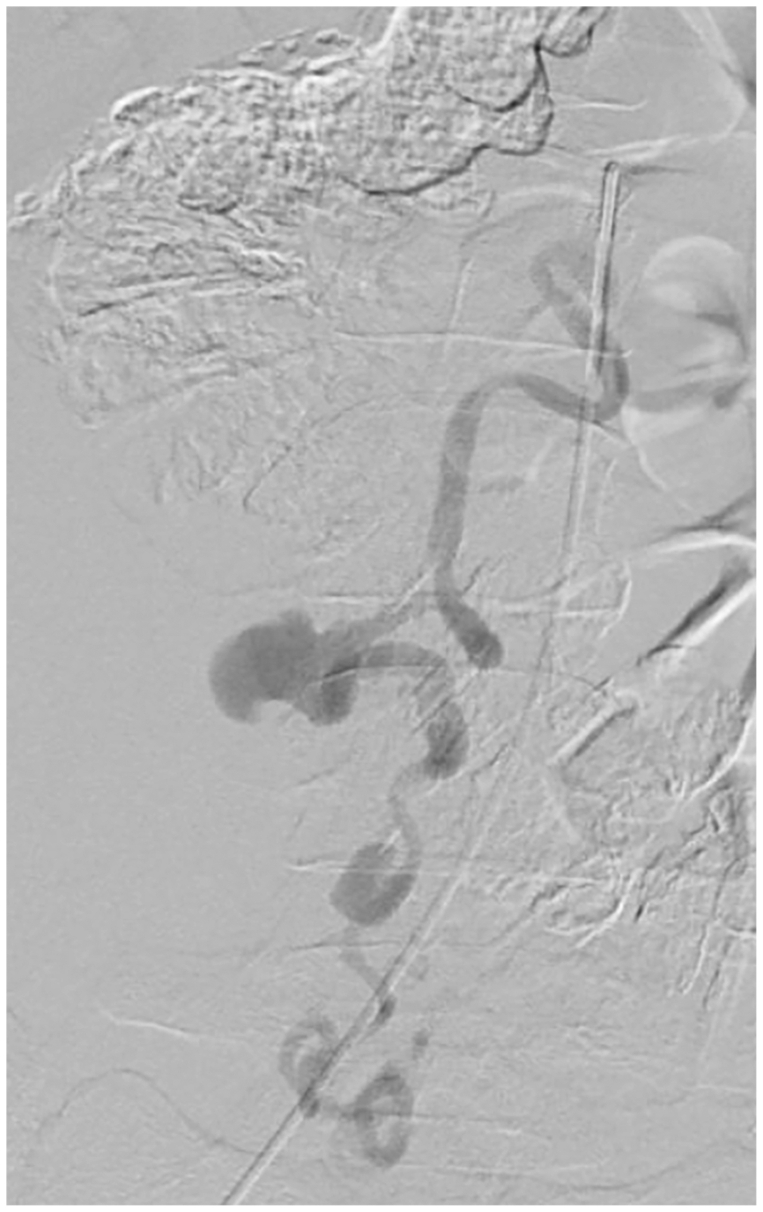
Right ovarian artery angiogram, demonstrating large aneurysm and significant tortuosity.

A 33-year-old woman with a history of a prior cesarean section and vaginal delivery 2 days prior presented with severe pelvic cramping and syncope. She was profoundly hypotensive with a systolic blood pressure of 60 mm Hg and a hemoglobin of 6.7 g/dL. After initial resuscitation, the patient underwent emergent dilation and curettage for presumed retained products of conception noted on transvaginal ultrasound examination. The patient remained hypotensive post procedure; a CT scan was obtained and demonstrated a large right retroperitoneal hematoma, with active extravasation of contrast from a 2.1-cm focal dilation of a tubular serpiginous vessel arising from the aorta, consistent with a ruptured aneurysm (Fig 1). The patient was then emergently transferred to the interventional radiology suite for intervention. From a 5F right common femoral artery access, bilateral uterine arteries were selectively cannulated using a UAC2 (Uterine Artery Catheter; Merit Medical Systems) and embolization of each was performed using a thrombin Gelfoam slurry (Pfizer), with technical success (Figs 2, *A, B*). The right ovarian artery was then cannulated at its orifice with an 0.018″ wire, and angiography demonstrated significant tortuosity with evidence of a distal right OAA (Fig 3) without obvious extravasation at the time. Embolization of this vessel was attempted in similar fashion to above, but the arterial tortuosity prevented catheter advancement. A Mikaelson catheter (Merit Medical Systems) was attempted without success. Thrombin could not be used due to the risk of systemic dissemination, and, even with selective cannulation with a 2.7F Progreat microcatheter (Terumo Interventional Systems), coils could not be advanced without losing access.

The decision was then made to abort further endovascular attempts, because they would likely continue to be unsuccessful, and to proceed with open surgical intervention to expeditiously control the bleeding. At this time, the patient had received numerous blood products, but remained hypotensive with a systolic blood pressure of 70 mm Hg and heart rate of >120 bpm. After induction with general anesthesia and intubation, a midline laparotomy incision was performed and the peritoneum was opened. A large right retroperitoneal hematoma was obvious. Mobilization of the visceral contents was achieved via a right medial visceral rotation (Cattell-Braasch maneuver), as well as completed Kocher maneuver of the duodenum. The right retroperitoneum was explored, and significant hematoma was evacuated. There was active bleeding noted, traced back to a long tortuous artery consistent with the angiographic findings of a ruptured right OAA, which was located close to its takeoff from the aorta, still within the retroperitoneal space. This was dissected free from surrounding structures and ligated proximally and distally with 2-0 silk ties. The gynecology team had been present during this case, and due to questionable viability of the right ovary, a right salpingo-oophorectomy was performed. Visceral contents were returned to anatomical locations and the abdomen was closed. The patient had an unremarkable postoperative course and rapidly stabilized following open intervention. The patient ultimately required 2 nights in the intensive care unit for monitoring, but required no further blood products or pressor support, and laboratory tests and vital signs all normalized. She was discharged home ambulatory on postoperative day 9. She has been seen at follow-up visits up to 1 year postoperatively and has healed well with no further hemorrhagic complications.

## Discussion

Ruptured OAA is a rare vascular emergency, and its infrequent presentation contributes to frequent diagnostic delay.^8^ Our patient's clinical course reflects this challenge. Her symptoms were intermittent, nonspecific, and overlapped with far more common postpartum conditions, resulting in an initially broad differential. Even once cross-sectional imaging was obtained, the diagnosis remained difficult because the large retroperitoneal hematoma obscured the small-caliber ovarian artery. Similar diagnostic ambiguity has been described in prior reports, where CT angiography failed to clearly identify the bleeding source due to vessel spasm, mass effect from the hematoma, or contrast dilution.^9^ This case reinforces that clinicians should maintain a high index of suspicion for visceral artery bleeding in postpartum patients with unexplained retroperitoneal hemorrhage.

Endovascular intervention is generally favored as the initial treatment for hemodynamically stable patients, given its minimally invasive nature and reported success with coil or particle embolization.^10^^,^^11^ Typically, uterine arteries are the targets for embolization initially, as this tends to manage >90% of postpartum bleeding, which is why this procedure was performed in this particular case.^12^ For embolization of the uterine arteries, fertility is often unaffected, but the rates are not known for that of ovarian arteries, as this is a seldom performed intervention^13^ However, published experiences also emphasize that endovascular failure is not uncommon, particularly in acutely ruptured OAAs or when vascular anatomy is unfavorable.^2^, ^3^, ^4^ Tortuosity, small vessel caliber, and challenging takeoff from the aorta may prohibit catheter stability and selective embolization. In our patient, these anatomical and technical limitations ultimately prevented adequate endovascular control. This result aligns with the literature, which notes that, although embolization is often effective, its success depends heavily on vascular access and visualization, both of which can be compromised in the presence of extensive retroperitoneal hemorrhage.^14^

There are no established, clear guidelines for the management of OAA; however, in the setting of ongoing bleeding and failed embolization, open surgical intervention became the appropriate next step. Operative exploration allows direct identification and control of the bleeding ovarian artery and facilitates evacuation of the hematoma, offering reliable and immediate hemostasis.^15^ Our patient stabilized rapidly after surgery, consistent with reports demonstrating that open repair remains the definitive and often life-saving treatment when endovascular options are either unsuccessful or not feasible. This approach has been seldom described in the literature in modern times, with the majority of cases managed endovascularly.^16^ This case highlights the importance of rapid transition from endovascular to open management when hemorrhage persists, because delays can lead to worsening hemodynamic instability.

## Conclusions

Ruptured OAA is an uncommon but potentially life-threatening cause of postpartum hemorrhage, often presenting with vague or intermittent symptoms that contribute to delayed diagnosis. Although endovascular embolization is an appropriate first-line therapy when anatomy permits, technical limitations can prevent successful occlusion. Our case highlights that rapid transition to open surgical management is essential when embolization fails, providing definitive hemorrhage control and timely clinical stabilization. This experience underscores the importance of multidisciplinary coordination and adds to the limited literature guiding treatment strategies for this rare vascular emergency.

## Funding

None.

## Disclosures

None.
